# The policy of control health and safety and the risk factors in the coal mining of east Kalimantan

**DOI:** 10.1186/1471-2458-14-S1-O26

**Published:** 2014-01-29

**Authors:** Krispinus Duma, Adi Heru Husodo, Soebijanto, Lientje Setyawati Maurits

**Affiliations:** 1 Bagian IKM, FK Universitas Mulawarman, Samarinda, Indonesia; 2 Bagian Ilmu Kesehatan Masyarakat, FK UGM, Yogyakarta 55281, Indonesia; 3 Bagian Anatomi, FK UGM, Yogyakarta 55281, Indonesia; 4 Program Studi Ilmu Kesehatan Kerja, FK UGM, Yogyakarta 55281, Indonesia

## Background

Occupation within the coal mining company is full of risks. Being located far from residential areas and the risk of occupational health and safety (HS) and the first-hand experience thereof may be considered to be limited to the miners themselves. The control policies by the government issued a once every three months visit to the main site. However, this is regarded as not being effective. Even though the supervision of the company's management during mining process takes place, but there are still a lot of work accidents. The purpose of this research was to design HS module booklet form as a list of assessment of the labour risk factors faced by working in the mines in the works for introspection despite minimal HS control policies.

## Materials and methods

This research employed an analytic quasi-experimental design, non-equivalent control group design approach, which consisted of the treatment group who received the intervention HS module and a control group without intervention. The difference in the output of the two groups was the effectiveness of the intervention module HS. The independent variables investigated were lifestyle, attitude-behaviour-knowledge of HS, and work environment factors with the variables fatigue and accidents. The study population was a heavy equipment operator coal mining company. Purposive sampling with random sampling of 592 operators was undertaken. Multivariate data analysis technique using Structural Equation Modeling with AMOS 7.0 program was employed.

## Results

The lifestyle was negatively correlated with the fatigue variables (-0.798, p<0.05). Behaviour-attitude-knowledge of HS was negatively correlated with fatigue (-0.762, p<0.05). Work environment factors were positively correlated with fatigue (0.791, p<0.05). Fatigue was positively correlated with occupational injuries (0.864, p<0.05).

## Conclusions

The present research shows that fatigue was significantly correlated with accidents in coalmines, thus require special attention of HS control policies from policy makers.

